# Algoriphagus aurantiacus sp. nov. and Algoriphagus persicinus sp. nov., two novel species isolated from the shore soil of salt lake

**DOI:** 10.1099/ijsem.0.006826

**Published:** 2025-06-27

**Authors:** Yan-Yan Zheng, Xuan Zhang, Zi-Xuan Liu, Rui Wang, Dorji Phurbu, Ai-Hua Li

**Affiliations:** 1 Xizang Key Laboratory of Plateau Fungi, Institute of Plateau Biology of Xizang Autonomous Region, Lhasa, Xizang Autonomous Region 850001, PR China; 2 China General Microbiological Culture Collection Center, Institute of Microbiology, Chinese Academy of Sciences, Beijing 100101, PR China; 3 School of Biotechnology and Food Science, Tianjin University of Commerce, Tianjin, PR China; 4 Tianjin Institute of Industrial Biotechnology, Chinese Academy of Sciences, Tianjin 300308, PR China

**Keywords:** *Algoriphagus*, genome, polyphasic taxonomy, salt lake, Xizang

## Abstract

Six Gram-staining-negative, aerobic, non-motile and rod-shaped bacterial strains, designated as D3-2-R+10^T^, C2-6-M1^T^, E1-3-M2, D2-2-M3, D2-2-M2 and D2-2-M1, were isolated from the shore soil of LungmuCo Lake in Xizang Autonomous Region, China. The 16S rRNA gene sequence comparisons confirmed that the six isolates belonged to the genus *Algoriphagus* of the family *Cyclobacteriaceae*. The 16S rRNA gene sequence of strains D3-2-R+10^T^, D2-2-M3, D2-2-M2 and D2-2-M1 exhibited the highest similarities to the type strains of *Algoriphagus antarcticus* LMG 21980^T^(98.1–98.5 %) and *Algoriphagus resistens* NH1^T^ (97.9–98.4 %). Meanwhile, the 16S rRNA gene sequence of strains C2-6-M1^T^ and E1-3-M2 showed the highest similarities to *A. antarcticus* LMG 21980^T^ (98.2–98.7%). The average nt identity and digital DNA–DNA hybridization values among strains D3-2-R+10^T^ and C2-6-M1^T^ and their most closely related species *A. antarcticus* LMG 21980^T^ were all lower than the threshold values for delineating species, indicating that they represent two separate novel species of *Algoriphagus*. The predominant cellular fatty acids of strains D3-2-R+10^T^ and C2-6-M1^T^ included iso-C_15 : 0_, summed feature 3 (C_16 : 1_* ω*7*c*/C_16 : 1_* ω6*c) and summed feature 9 (iso-C_17 : 1_* ω*9*c*/C_16 : 0_ 10-methyl), and the predominant respiratory quinone was MK-7. The major polar lipids of strain D3-2-R+10^T^ comprised phosphatidylethanolamine, one unidentified phospholipid and five unidentified lipids, while those of strain C2-6-M1^T^ included phosphatidylethanolamine, two unidentified phospholipids and seven unidentified lipids. The genome size of strains D3-2-R+10^T^ and C2-6-M1^T^ was 6.0 and 5.0 Mb, respectively, with the DNA G+C contents of 40.1 and 40.9 mol %. Based on the above descriptions, the six strains were identified as two novel species of the genus *Algoriphagus*, for which the names *Algoriphagus aurantiacus* sp. nov. and *Algoriphagus persicinus* sp. nov. were proposed, with the type strains, respectively.

## Introduction

The genus *Algoriphagus* belongs to the family *Cyclobacteriaceae* within the phylum *Bacteroidota*. This genus was first proposed by Bowman *et al*. [1], with *Algoriphagus ratkowskyi* designated as the type species. Members of the genus *Algoriphagus* are Gram-stain-negative and non-motile and possess MK-7 as the predominant respiratory quinone [2]. As of this writing, the genus *Algoriphagus* comprises 50 species with validly published names (https://lpsn.dsmz.de/search?word=Algoriphagus). The species of the genus *Algoriphagus*, which were widely distributed in marine environments, such as *A. ratkowskyi*, isolated from sea ice and saline lake cyanobacterial mats, is a cold-adapted strain exhibiting optimal growth below 22 °C and sustained growth at −2 to −1 °C. This psychrophilic bacterium represents a promising source of cold-adaptation enzymes [1]: *Algoriphagus antarcticus*, a psychrophile isolated from microbial mats in Antarctic lakes, which was most closely related to the genera *Hongiella*, *Belliella* and *Cyclobacterium* [3]; *Algoriphagus locisalis*, isolated from sea water collected from a marine solar saltern of the Yellow Sea, Korea [4]; *Algoriphagus faecimaris*, isolated from coastal sediment of Jiaozhou Bay, near the estuary of the river Lichun at Qingdao, China, on the coast of the Yellow Sea [5]; *Algoriphagus hitonicola*, isolated from salty water from the athalassohaline lagoon at El Hito, located in central Spain [6]; *Algoriphagus iocasae*, isolated from the deep-sea sediment of the Okinawa Trough, obtained by a remotely operated vehicle equipped on [7]; *Algoriphagus lutimaris*, isolated from tidal flat sediment on the west coast of Korea [8]; *Algoriphagus resistens*, isolated from marine sediment along the coast of Weihai, China [9]; and so on. It could be seen that the vast majority of strains of the genus *Algoriphagus* were isolated from marine environments or hypersaline environments. Recently, bioinformatic analyses showed that ~69% of bacteria in the *Algoriphagus* genus harboured a putative contractile injection system (CIS) gene cluster, which were phage tail-like nanomachines, mediating bacterial cell–cell interactions as either type VI secretion systems or extracellular CISs [10].

In this study, six orange–red-coloured or pink-coloured strains were isolated from the shore soil of LungmuCo Lake in Xizang Autonomous Region, China. Preliminary analysis of the 16S rRNA gene sequences indicated that strains D3-2-R+10^T^ and C2-6-M1^T^ represent two potential novel species within the genus *Algoriphagus*. The aim of this study was to establish the taxonomic position of these two bacterial strains by using a polyphasic approach that included the determination of phenotypic and chemotaxonomic properties, a detailed phylogenetic investigation based on 16S rRNA gene sequences and genetic analysis.

## Methods

### Isolation

LungmuCo Lake, a saline lake located in the Ali region of Xizang, China, has an altitude of 5,030 m and experiences an annual average temperature range of −6 to 9 °C. Due to intense evaporation along its shore, the lakeside soil is predominantly saline with a high salinity exceeding 17%. Soil samples for microbial strain isolation were collected from this saline shoreline environment in August 2020 (coordinates: 34° 33′ N 80° 23′ E), during the investigation of the bacterial biodiversity of salt lakes in the region. Samples were collected from beneath the surface at a depth of ~20–30 cm.

For the isolation process, 10 g of soil was suspended with 90 ml of sterile 0.5% NaCl solution, followed by serial tenfold dilution with the same solution and thorough mixing. Samples were vibrated and mixed evenly before each dilution, after which a 200 µl solution was spread onto three types of agar plates: marine 2216 agar (MA; Difco, Becton Dickinson), R2A and R2A plus 0.5% NaCl. All plates were incubated at 15 °C for 2 weeks. Single colonies were selected and further purified. All the purified strains were preserved through lyophilization and liquid nitrogen.

### 16S rRNA gene phylogeny

The 16S rRNA gene sequences of the isolates were amplified using the universal primers 27F and 1492R [11,12]. The complete 16S rRNA gene sequences were aligned via the EzBioCloud database (https://www.ezbiocloud.net/) and GenBank database [13]. Further investigation of genotypic characteristics was performed by phylogenetic analysis. The 16S rRNA gene sequences of the isolates and those of related taxa retrieved from the GenBank database were aligned with BioEdit [14]. Phylogenetic trees were reconstructed using neighbour-joining [15], maximum-likelihood [16] and minimum-evolution [17] methods in mega 7.0 [18]. Evolutionary distances for the neighbour-joining tree were calculated by Kimura’s two-parameter model [19]. The tree topologies were evaluated by bootstrap analysis based on 1,000 resamplings of the data.

### Phylogenomic analysis and genomic annotation

For genome sequence analysis, the total DNA of strains D3-2-R+10^T^ and C2-6-M1^T^ was extracted using the Genomic DNA Rapid Isolation Kit (BioDev-Tech). Genome sequencing was performed on the Illumina HiSeq X platform at Majorbio Science and Technology Ltd. The quality of raw data (genome 100 coverage depth) was analysed and controlled using statistical methods. The clean data were assembled from scratch with SOAP *de novo* (v2.0; http://soapdenovo2.sourceforge.net/) to get the optimal assembly results [20,21]. The contamination values of the genome sequence were assessed using CheckM [20]. The coding sequence (CDS) prediction was conducted using Glimmer (v3.02; http://ccb.jhu.edu/software/glimmer/index.shtml) and GeneMarkS (v4.3; http://topaz.gatech.edu/GeneMark). The rRNA and tRNA were predicted using Barrnap (v0.8; https://github.com/tseemann/barrnap/) and tRNA-scan-SE (v2.0; http://trna.ucsc.edu/software/), respectively.

To further confirm the phylogenomic position of the isolates within the genus *Algoriphagus*, a phylogenomic tree was reconstructed by using Type Strain Genome Server (https://tygs.dsmz.de) [22]. The average nt identity (ANI) was calculated by using EzGenome (http://www.ezbiocloud.net/genome/list?tn=Root). The average aa identity (AAI) was determined using the Kostas lab (http://enve-omics.ce.gatech.edu/aai/). The digital DNA–DNA hybridization (dDDH) values between the novel isolates and the reference species were calculated using the Genome-to-Genome Distance Calculator (GGDC 2.1) (http://ggdc.dsmz.de/ggdc.php) [23].

The pan-genome analysis was performed using PGAP (v1.2.1; https://jaist.dl.sourceforge.net/project/pgap/PGAP) and GETHOMOLOGUS (v3.2.3; https://github.com/eead). Genes present in all input genomes were categorized as core genes, while single genes found in only one genome were classified as unique genes, and the remaining genes were classified as dispensable genes. The genes were organized into subsystems and clusters of orthologous groups (COGs) using the Rapid Annotation using Subsystem Technology (RAST) server (https://rast.nmpdr.org/) [24]. Genomic annotation was carried out using the NR (ftp://ftp.ncbi.nlm.nih. gov/blast/db/), KEGG (http://www.genome.jp/kegg/) and eggNOG (http://eggnogdb.embl.de/#/app/home), respectively. The antiSMASH was employed to predict biosynthetic gene clusters of secondary metabolites (https://dl.secondarymetabolites.org/releases/4.0.2/).

### Phenotypic characteristics

Cell morphology and flagella were observed using light microscopy and transmission electron microscopy (JEM1400, JEOL). The Gram-staining reaction was performed according to standard protocol [25]. Motility was determined using the hanging-drop method, and gliding motility was determined as described by Bowman [26]. NaCl tolerance was determined in R2A broth medium with NaCl concentrations ranging from 0 to 17% (w/v, 0, 0.5 and 1%, with the remaining salt concentration intervals at 2%) and incubated at 25 °C for 2 weeks. Growth at various temperatures (4, 10, 15, 20, 25, 30, 35 and 40 °C) for 2 weeks was assessed in marine 2216 broth (MB) with the optimal NaCl concentration. MB medium with different pH (5.0–11.0, at 0.5 pH unit intervals) was prepared to determine the pH range for growth using the corresponding buffers: 100 mM citric acid/sodium citrate buffer (for pH 5.0–5.5), 200 mM phosphate buffer (for pH 6.0–8.0), 50 mM Trizma base/Trizma HCl buffer (for pH 8.5) or 200 mM NaHCO_3_/Na_2_CO_3_ buffer (for pH 9.0–10.0). Strains were cultured in broth at 25 °C for 2 weeks. Catalase activity was assessed via observing bubble production in a reaction with 5.0% (v/v) H_2_O_2_. The oxidase activity test was conducted using the Bactident Oxidase Strips, Merck detection kit. Growth under anaerobic conditions was determined by cultivating on MA plates with or without 0.1% (w/v) KNO_3_ in an anaerobic chamber with anaeropacks for 2 weeks at 25 °C. Substrate degradation was evaluated on MA plates, adding 1.0% Tween 20, 1.0% Tween 80, 1.0% casein, 1.0% starch, 1.0% sodium carboxymethyl cellulose, 1.0% urea and 1.0% gelatin (w/v), respectively [27]. Transparent circles appearing around the colonies on the plate were observed after 7-day incubation. Additional enzyme activities, assimilation and acid production were examined using API ZYM, API 20NE, API 20E and API 50CH strips (bioMérieux), following the manufacturer’s instructions. The utilization of sole carbon and nitrogen sources was determined using a Biolog GEN III MicroStation system following the manufacturers’ instructions. Photosynthetic pigments were extracted from cells grown in MB at 25 °C in the dark. Acetone/methanol (7:2) was employed to extract the pigments, and the absorption spectrum was recorded from 300 to 900 nm by spectrophotometer as outlined by Biebl *et al*. [28].

For the analysis of cellular fatty acids, quinones and polar lipids, strains D3-2-R+10^T^ and C2-6-M1^T^ were cultivated in MB at 25 °C and harvested during the late exponential phase. Cellular masses were collected by centrifugation and subsequently saponified, methylated and extracted according to the standard protocol in the MIDI/Hewlett Packard Microbial Identification System [29]. The extraction was then analysed via a gas chromatograph (6890 N; Agilent) and identified through the TSBA 6.0 database [30]. The menaquinones and polar lipid profiles were analysed according to the method of Minnikin *et al*. [31]. Isoprenoid quinone was analysed using an HPLC. The polar lipid was separated using two-dimensional TLC and then identified by spraying molybdatophosphoric acid (for total lipids), phosphomolybdic acid (for phospholipids), ninhydrin (for aminolipids) and *α*-naphthol/sulphuric acid reagent (for glycolipids), respectively [31].

Antibiotic sensitivity was tested on MA plates using antibiotic discs (Beijing Tiantan Biological Products) containing fleroxacin (5 µg), lomefloxacin (10 µg), ciprofloxacin (5 µg), penicillin (10 IU), erythromycin (15 µg), chloramphenicol (30 µg), azithromycin (15 µg), clindamycin (2 µg), doxycycline (30 µg), clarithromycin (15 µg), tobramycin (10 µg), vancomycin (30 µg), netilmicin (30 µg), ceftriaxone (30 µg), cefaclor (30 µg), cefazolin (30 µg), cefotaxime (30 µg), ampicillin (10 µg), cefuroxime sodium (30 µg), minocycline (30 µg), rifampin (5 µg), tetracycline (30 µg), sulfamethoxazole/trimethoprim (1.25 µg), amikacin (30 µg), ceftazidime (30 µg), cephalotin (30 µg), cefoperazone (75 µg), piperacillin (100 µg), oxacillin (1 µg) and nitrofurantoin (300 µg). All plates were incubated at 25 °C for 7 days.

## Results and discussion

### Bacterial isolation

This investigation into bacterial biodiversity isolated ~1,005 strains. Clear differences were observed among soil, water and sediment samples*. Halomonas*, *Roseovarius* and Psychroflexus dominated the lake water samples, while *Marinobacter*, *Lutimonas* and *Gracilimonas* were the dominant groups in the sediment. The abundances of *Truepera* and *Lysobacter* were significantly higher in both soil and sediment samples compared with lake water.

### Phylogenetic and phylogenomic analysis

**Fig. 1.**
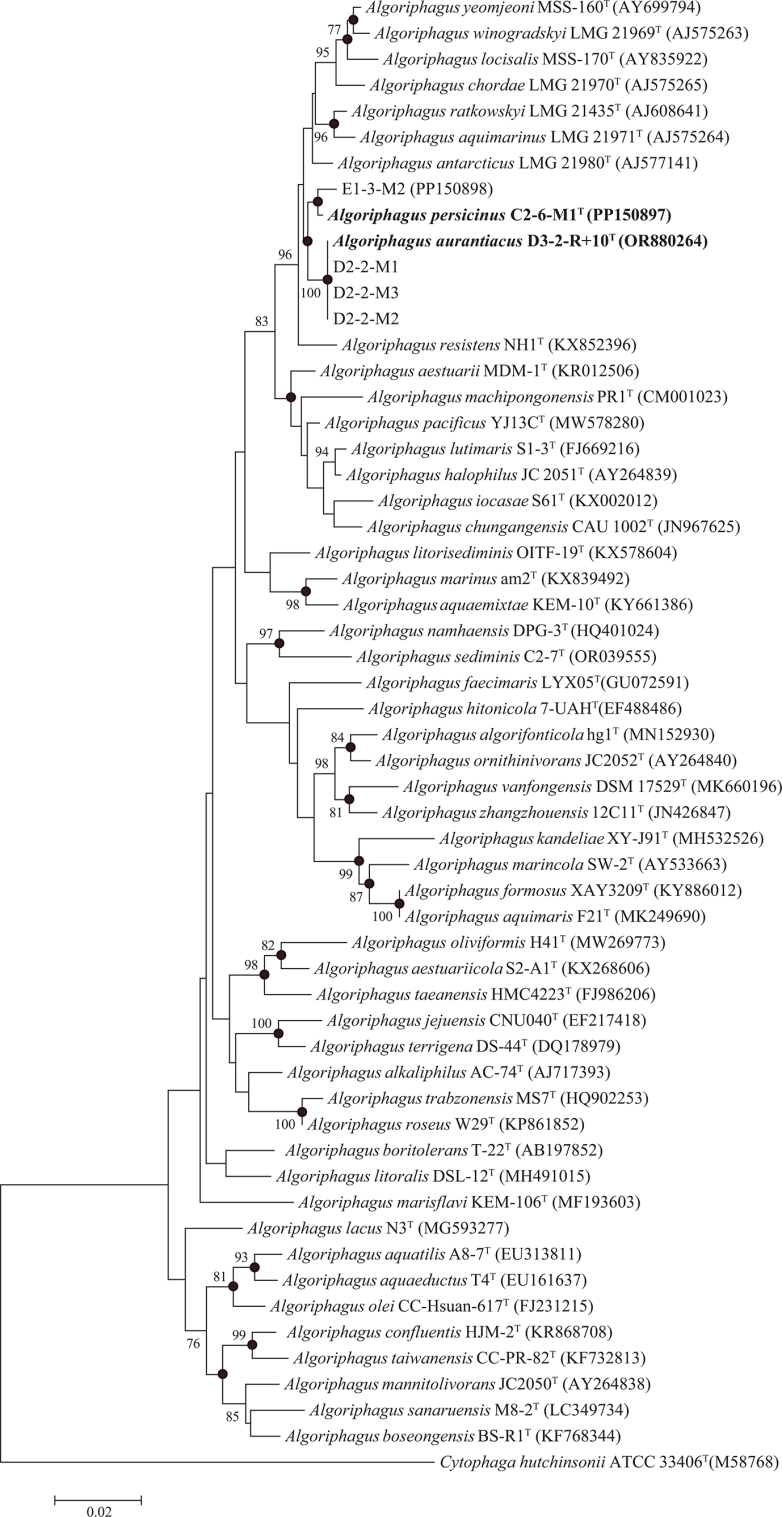
The neighbour-joining tree reconstructed based on 16S rRNA gene sequences of strains D3-2-R+10^T^, C2-6-M1^T^, E1-3-M2, D2-2-M3, D2-2-M2 and D2-2-M1 and type species of the genus *Algoriphagus*. Bootstrap values higher than 70% are shown (percentages of 1,000 replications) on the branches. Filled circles indicate that the corresponding nodes were also present in the trees reconstructed using the maximum-likelihood and minimum-evolution algorithms. Bar represents 0.02 changes per nt position. *Cytophaga hutchinsonii* ATCC 33406^T^ was utilized as an outgroup.

Based on the analysis of 16S rRNA gene sequences, strains D3-2-R+10^T^, D2-2-M3, D2-2-M2 and D2-2-M1 showed 99.9% similarity in pairwise comparisons, indicating that they represent the same species. Strain D3-2-R+10^T^ was chosen for further analysis. C2-6-M1^T^ and E1-3-M2 shared 99.3% similarity, suggesting that they may represent the same species. Strains D3-2-R+10^T^ and C2-6-M1^T^ shared a pairwise similarity of 99.1%. 16S rRNA gene sequences extracted from whole-genome assemblies showed high concordance with Sanger sequencing results: 99.78% for strain D3-2-R+10^T^ and 99.93% for strain C2-6-M1^T^. blast alignment revealed that the similarity of 16S rRNA gene sequences extracted from the genome of strains D3-2-R+10^T^ and C2-6-M1^T^ showed the highest similarity (98.71% and 98.64%) to *A. antarcticus* LMG 21980^T^, respectively. The phylogenetic trees based on 16S rRNA gene sequences of the novel strains and all validly published type species of *Algoriphagus* were constructed using neighbour-joining, maximum-likelihood and minimum-evolution methods. The neighbour-joining tree demonstrated that strains D3-2-R+10^T^, D2-2-M3, D2-2-M2 and D2-2-M1 formed a clade, which then grouped with the clade formed by strains C2-6-M1^T^ and E1-3-M2 and subsequently clustered with other species of *Algoriphagus* (Fig. 1). The result was further supported by the phylogenetic trees reconstructed using the maximum-likelihood and minimum-evolution algorithms, respectively (Figs S1 and S2, available in the online Supplementary Material).

**Fig. 2.**
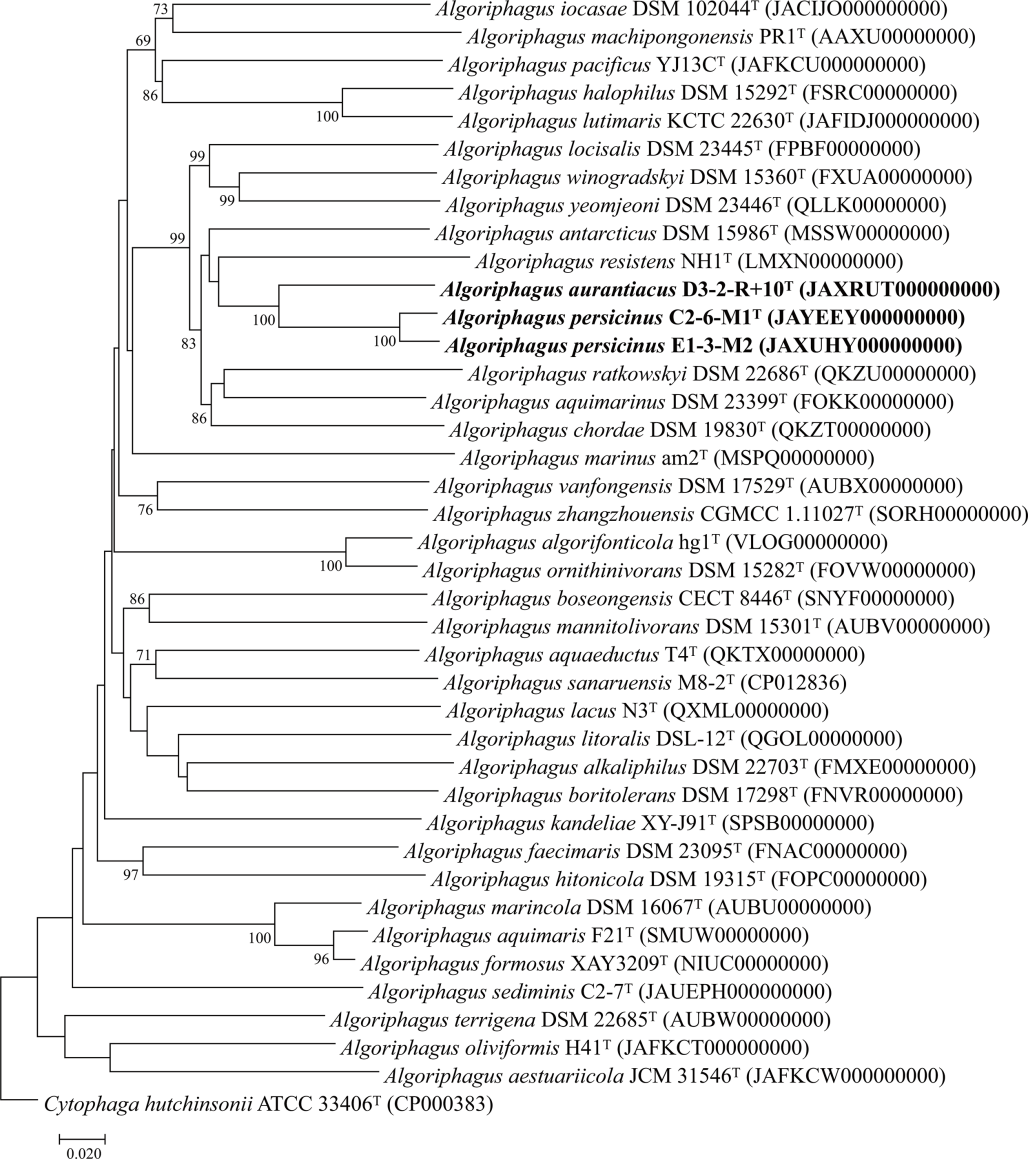
The phylogenomic tree based on the genomic sequences of strains D3-2-R+10^T^ and C2-6-M1^T^ and type strains of the related species constructed using TYGS. *Cytophaga hutchinsonii* ATCC 33406^T^ was utilized as an outgroup.

To strengthen the phylogenetic status and better characterize the relationships of the novel isolates, a phylogenomic tree was reconstructed based on the genomic sequences of all novel isolates and type strains of the related species. Strains D3-2-R+10^T^ and C2-6-M1^T^ were not classified within any valid species in the genus *Algoriphagus*, reinforcing the conclusion that these represent two novel species of the genus *Algoriphagus* (Fig. 2). The ANI, AAI and dDDH values between strains C2-6-M1^T^ and E1-3-M2 were 96.7%, 97.1% and 71.8%, respectively, showing that they belonged to the same species of the genus *Algoriphagus*. Additionally, the ANI, AAI and dDDH values between strains D3-2-R+10^T^ and C2-6-M1^T^ were 86.9%, 89.4% and 32.6%, respectively. And the ANI, AAI and dDDH values of strains D3-2-R+10^T^ and C2-6-M1^T^ with their most closely related reference strain *A. antarcticus* DSM 15986^T^ were 81.2% and 81.1% (ANI), 85.3% and 85.2% (AAI) and 24.5% and 24.5% (dDDH), respectively, all falling below the threshold values of 95–96% ANI/AAI and 70% dDDH for species delineation [32,33], indicating that they represent two different novel species of the genus *Algoriphagus* (Table 1).

**Table 1.** ANI and dDDH values between strains D3-2-R+10^T^, C2-6-M1^T^ and E1-3-M2 and related type strains of species of the genus *Algoriphagus* Strains: 1, D3-2-R+10^T^; 2, C2-6-M1^T^; 3, E1-3-M2; 4, *A. antarcticus* DSM 15986^T^; 5, *A. ratkowskyi* DSM 22686^T^; 6, *A. resistens* NH1^T^. The GenBank accession numbers for genomes of the six strains used for ANI and dDDH analysis were AXRUT000000000, JAYEEY000000000, JAXUHY000000000, MSSW00000000, QKZU00000000 and LMXN00000000, respectively.

**ANI**						
**dDDH**	1	2	3	4	5	6
**1**	−	86.9	86.6	81.2	78.9	80.3
**2**	32.6	−	96.7	81.1	79.0	80.1
**3**	32.4	71.8	−	81.1	79.1	79.9
**4**	24.5	24.5	24.5	−	80.3	79.2
**5**	22.2	22.2	22.4	23.4	−	77.5
**6**	23.6	23.4	23.2	22.8	20.8	−

### Phenotypic characteristics

**Fig. 3.**
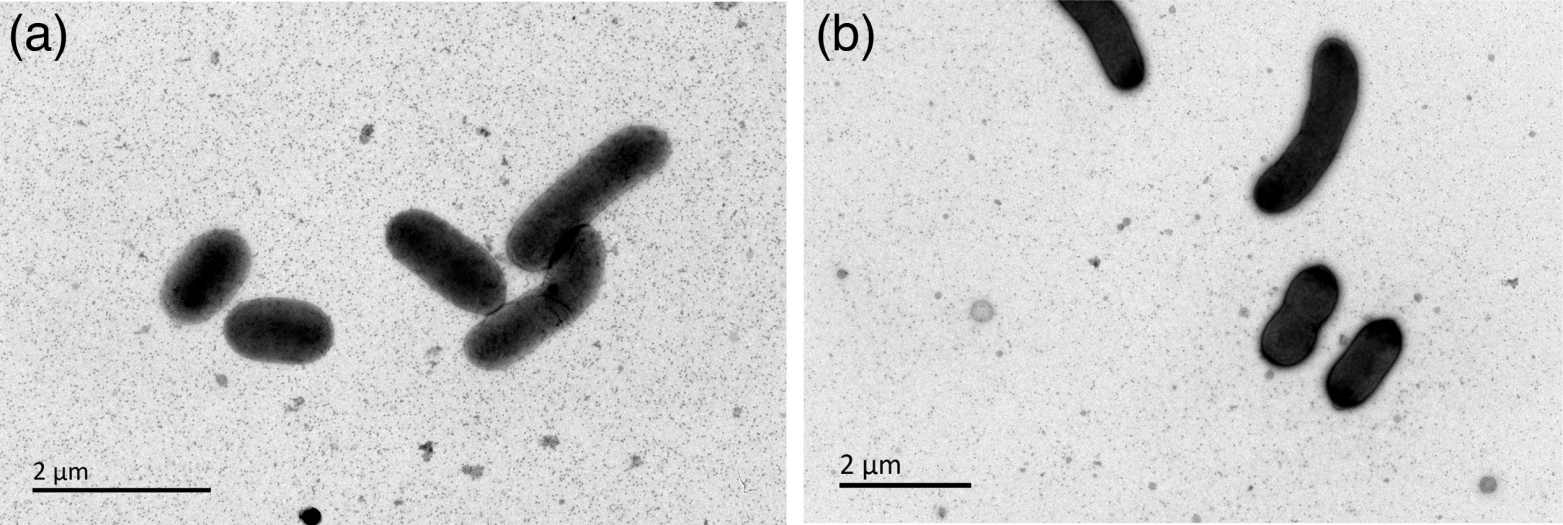
Transmission electron micrograph of strains D3-2-R+10^T^ (**a**) and C2-6-M1^T^ (**b**) cultivated on MA plate at 25 °C for 72 h.

**Fig. 4.**
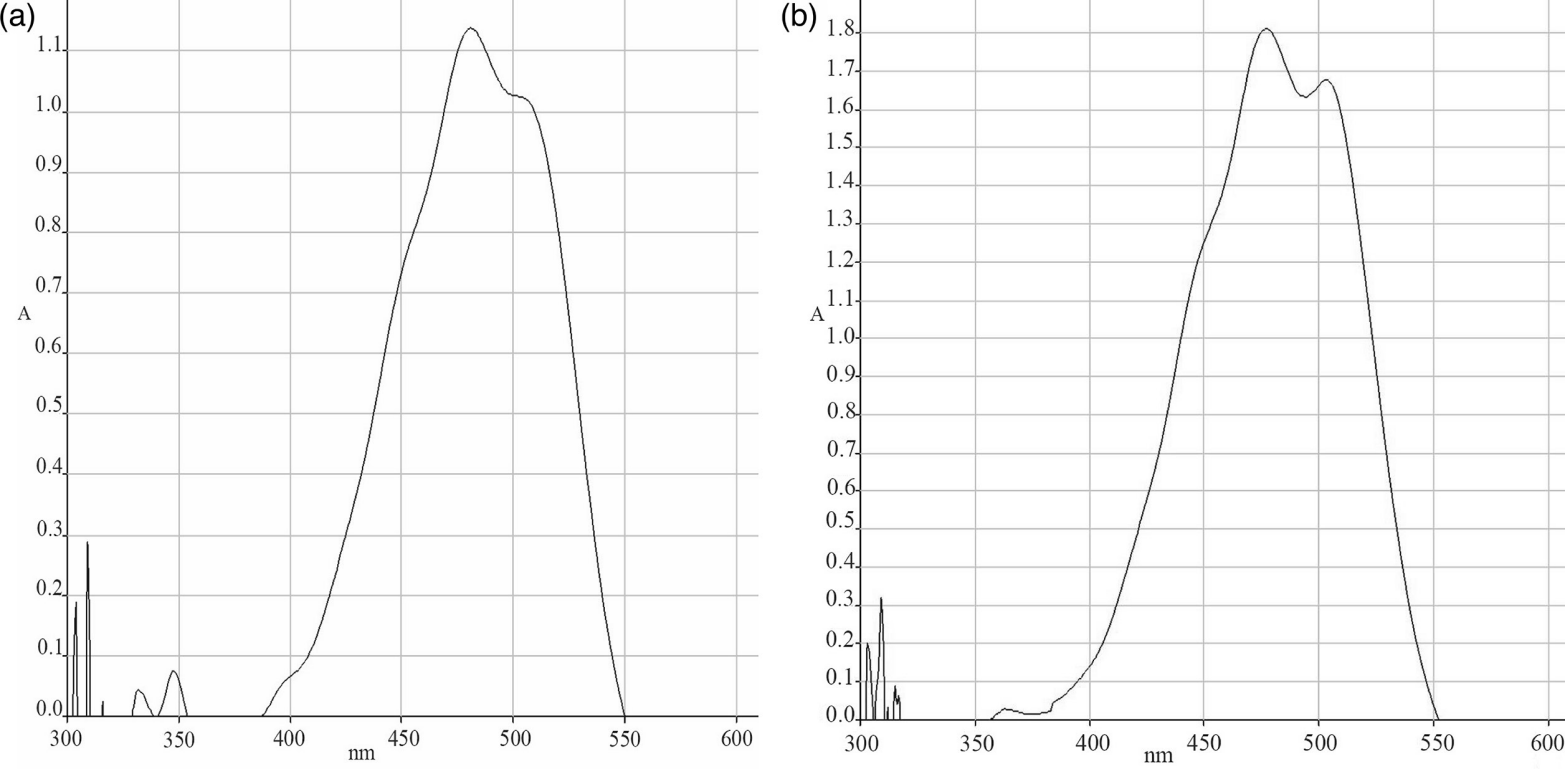
Absorption spectra of acetone/methanol cell extract from strains D3-2-R+10^T^ (**a**) and C2-6-M1^T^ (**b**) in the range of 300–900 nm. The absorbance peak at ~480 nm indicates the presence of carotenoids. The horizontal coordinate is wavelength, and the vertical coordinate is absorbance.

Strains D3-2-R+10^T^ and C2-6-M1^T^ were Gram-stain-negative, aerobic, non-motile and catalase- and oxidase-positive. After 3 days of incubation on MA plates at 25 °C, colonies were convex, circular, smooth, opaque and orange–red-pigmented (for strain D3-2-R+10^T^) or pink-pigmented (for strain C2-6-M1^T^). The cell size of strain D3-2-R+10^T^ was 0.6–0.8 µm in width and 1.2–2.4 µm in length when observed under the transmission electron microscope (JEM1400, JEOL), and that of C2-6-M1^T^ was 0.6–0.8 µm in width and 1.2–2.2 µm in length (Fig. 3). The growth of strain D3-2-R+10^T^ occurred at 4–30 °C (optimum, 25 °C) and pH 5.0–8.5 (optimum, pH 7.0), with NaCl concentrations from 0.5 to 13.0% (optimum, 5.0–7.0%). The growth of strain C2-6-M1^T^ occurred at 4–30 °C (optimum, 20 °C) and pH 5.0–8.0 (optimum, pH 7.0), with NaCl concentrations ranging from 0.5 to 15.0% (optimum, 5.0–7.0%). Strains D3-2-R+10^T^ and C2-6-M1^T^ synthesized carotenoid, as indicated by the absorption at 450–550 nm in the near IR spectrum of an acetone/methanol cell extract (Fig. 4). Detailed results of phenotypic and physiological properties are summarized in Table 2 and in the novel species description (Tables 2 and S1).

**Table 2.** Comparison of characteristics between novel isolates and closely related species of the genus *Algoriphagus* Strains: 1, D3-2-R+10^T^; 2, C2-6-M1^T^; 3, *A. antarcticus* CCTCC AB 2014250^T^; 4, *A. ratkowskyi* CCTCC AB 2010233^T^; 5, *A. resistens* NH1^T^. Unless otherwise specified, all data were from our experiments. O, orange; R, red; P, pink; +, positive; −, negative; w, weakly positive; nd, no data available.

Characteristic	1	2	3	4	5*
**Colour of cell mass**	O–R	P	O–R	P	P
**Temperature range for growth (optimal, °C)**	4–30 (25)	4–30 (20)	5–25 (20)	−2 to 25 (16–19)	13–37 (28)
**NaCl range for growth(optimal, %, w/v)**	0.5–13.0 (5.0–7.0)	0.5–15.0 (5.0–7.0)	0.0–5.0 (nd)	0.5–6.0 (1.0–2.0)	0.0–8.0 (3.0)
**pH range for growth (optimal pH)**	5.0–8.5 (7.0)	5.0–8.0 (7.0)	nd	6.5–8.5 (7.5–8.0)	5.5–8.5 (6.5–7.0)
**Hydrolysis of:**					
Tween 20	+	+	−	+	−
Tween 40	+	−	−	+	−
Tween 80	+	+	−	−	−
**Nitrate reduction**	+	+	−	−	+
**Production of:**					
Catalase	+	+	+	−	+
Oxidase	+	+	+	+	−
**Acid production from: (API 50 CH)**					
l-Arabinose, trehalose	+	+	−	+	−
Cellobiose	+	w	−	+	+
d-Fructose, d-galactose	+	+	−	+	−
d-Glucose, d-mannose, maltose	+	+	−	+	+
d-Xylose	+	w	−	+	+
**Utilization of: (from Biolog GEN III)**					
d-Galactose	w	+	+	+	+
d-Glucose, maltose, d-mannose	+	+	−	+	+
d-Mannitol	w	w	−	+	−
Sorbitol	w	w	+	+	−
Glycerol	−	w	−	−	−
**Enzyme activity (API ZYM):**					
Valine arylamidase, trypsin	+	+	+	−	+
Acid phosphatase	+	+	−	−	+
*β*-Galactosidase	+	+	−	+	−
*α*-Glucosidase	+	+	+	−	−
*β*-Glucosidase	+	+	+	+	−

*Data are from Han *et al.* [9].

The major respiratory quinone for strains D3-2-R+10^T^ and C2-6-M1^T^ was MK-7, consistent with the quinone profile of the genus *Algoriphagus*. The major polar lipids for strain D3-2-R+10^T^ contained phosphatidylethanolamine (PE), one unidentified phospholipid (PL) and five unidentified lipids (L1–L5). The major polar lipids for strain C2-6-M1^T^ contained PE, two PLs (PL1–PL2) and seven unidentified lipids (L1–L7) (Fig. S3). The major cellular fatty acids of strain D3-2-R+10^T^ were iso-C_15 : 0_ (33.2%), summed feature 3 (C_16 : 1_* ω7*c and/or C_16 : 1_* ω6*c; 16.2%) and summed feature 9 (C_16 : 0_ 10-methyl and/or iso-C_17 : 1_* ω9*c; 13.4%). The major cellular fatty acids of strain C2-6-M1^T^ were iso-C_15 : 0_ (29.0%), summed feature 3 (C_16 : 1_* ω7*c*/*C1_6 : 1_* ω6*c; 22.3%) and summed feature 9 (C_16 : 0_ 10-methyl/iso-C_17 : 1_* ω9*c; 8.7%). The contents of iso-C_15 : 0_ and summed feature 9 (C_16 : 0_ 10-methyl/iso-C_17 : 1_* ω*9*c*) of strain D3-2-R+10^T^ (33.2%, 13.4%) were higher than C2-6-M1^T^ (29.0%, 8.7%), while the content of summed feature 3 (C_16 : 1_* ω7*c*/*C1_6 : 1_* ω6*c; 16.2%) of strain D3-2-R+10^T^ (16.2%) was lower than C2-6-M1^T^ (22.3%). Furthermore, it was evident that the content of iso-C_15 : 0_ of strains D3-2-R+10^T^ and C2-6-M1^T^ (33.2%, 29.0%) was higher than their three relative species (*A. antarcticus* CCTCC AB 2014250, 20.4%; *A. ratkowskyi* CCTCC AB 2010333^T^, 17.9%; and *A. resistens* NH1^T^, 19.2%), while the content of summed feature 3 (C_16 : 1_* ω7*c and/or C_16 : 1_* ω6*c) of strain D3-2-R+10^T^ (16.2%) was lower than their reference strains (*A. antarcticus* CCTCC AB 2014250^T^, 17.2%; *A. ratkowskyi* CCTCC AB 2010333^T^, 24.0%; and *A. resistens* NH1^T^, 33.6%); the content of summed feature 3 (C_16 : 1_* ω7*c*/*C1_6 : 1_* ω6*c) of strain C2-6-M1^T^ (22.3%) was higher than that of *A. antarcticus* CCTCC AB 2014250 ^T^ (17.2%) and lower than that of *A. ratkowskyi* CCTCC AB 2010333 ^T^ (24.0%) and *A. resistens* NH1^T^ (33.6%) (Table 3). Based on the antibiotic sensitivity test, it was evident that strain D3-2-R+10^T^ was resistant to penicillin (10 IU), tobramycin (10 µg), netilmicin (30 µg), ceftriaxone (30 µg), cefotaxime (30 µg), cefuroxime sodium (30 µg), minocycline (30 µg), sulfamethoxazole/trimethoprim (1.25 µg), amikacin (30 µg), ceftazidime (30 µg), cephalotin (30 µg) and oxacillin (1 µg), and strain C2-6-M1^T^ was resistant to tobramycin (10 µg), netilmicin (30 µg), ceftriaxone (30 µg), cefazolin (30 µg), cefotaxime (30 µg), cefuroxime sodium (30 µg), sulfamethoxazole/trimethoprim (1.25 µg), amikacin (30 µg), ceftazidime (30 µg), cephalotin (30 µg) and oxacillin (1 µg) (Table S1).

**Table 3.** Cell fatty acid profiles of novel strains and type strains of the related *Algoriphagus* species Strains: 1, D3-2-R+10^T^; 2, C2-6-M1^T^; 3, *A. antarcticus* CCTCC AB 2014250^T^; 4, *A. ratkowskyi* CCTCC AB 2010233^T^; 5, *A. resistens* NH1 ^T^. Only fatty acids amounting to at least 1.0% of the total cellular fatty acids of at least one of the strains are shown. −, Not detected; tr, trace (<1.0 %); nd, no data available.

Fatty acid	1	2	3	4	5†
**Straight chain:**					
C_16 : 0_	−	−	1.1	1.9	1.8
**Unsaturated**					
C_15 : 1_* ω*6*c*	tr	1.4	1.0	2.3	tr
C_16 : 1_* ω*5*c*	2.2	1.7	6.4	8.4	7.1
**Branched**					
Anteiso-C_11 : 0_	1.6	−	nd	nd	nd
Iso-C_14 : 0_	1.7	3.0	tr	1.4	tr
Iso-C_15 : 1_ G	6.0	2.3	15.7	7.9	4.3
Iso-C_15 : 0_	33.2	29.0	20.4	17.9	19.2
Anteiso-C_15 : 0_	1.7	3.1	4.5	6.2	1.8
Iso-C_16 : 0_	1.8	2.4	3.5	4.1	3.3
Iso-C_16 : 1_ h	4.0	7.4	3.6	4.9	4.5
**Hydroxy**					
Iso-C_15 : 0_ 3-OH	4.0	2.8	2.6	1.7	2.2
Iso-C_16 : 0_ 3-OH	tr	1.3	1.5	1.7	tr
Iso-C_17 : 0_ 3-OH	4.3	3.7	6.1	4.2	3.0
Summed features*					
3	16.2	22.3	17.2	24.0	33.6
4	1.9	2.2	1.3	1.1	1.0
9	13.4	8.7	nd	nd	nd

*Summed features are fatty acids that cannot be resolved reliably from another fatty acid using the chromatographic conditions chosen. The MIDI system groups these fatty acids together as one feature with a single percentage of the total. Summed feature 3, C_16 : 1_* ω*6*c* and /or C_16 : 1_* ω*7*c*; summed feature 4, iso-C_17 : 1_ I and /or anteiso-C_17 : 1_ B; summed feature 9, C_16 : 0_ 10-methyl and /or iso-C_17 : 1_* ω*9*c*.

†Data are from Han *et al*. [9].

### Genome features

The completeness and contamination indices of the genome of strain D3-2-R+10^T^ were assessed as 99.16% and 5.51 %, respectively, and those of strain C2-6-M1^T^ were 97.63% and 2.07 %, respectively. Strain D3-2-R+10^T^ generated 1.2 Gb of clean bases, and the genome size was 6.0 Mb, which was assembled into 90 scaffolds with an N50 value of 171,452 bp. A total of 5,320 genes were predicted, including 5 rRNA genes (three 5S rRNA genes, one 16S rRNA gene and one 23S rRNA gene) and 43 tRNA genes. The genomic DNA G+C content of strain D3-2-R+10^T^ was 40.1%, calculated from the genome sequence data. Strain C2-6-M1^T^ generated 1.2 Gb of clean bases, and the genome size was 5.0 Mb with a DNA G+C content of 40.9%. It contained 78 scaffolds with an N50 of 164,373 bp. A total of 4,410 genes were predicted, including 4 rRNA and 43 tRNA (Table 4).

**Table 4.** The genomic features of strains D3-2-R+10^T^ and C2-6-M1^T^ and their related phylogenetic neighbours of *Algoriphagus* Strains: 1, D3-2-R+10^T^; 2, C2-6-M1^T^; 3, *A. antarcticus* DSM 15986^T^; 4, *A. ratkowskyi* DSM 22686^T^; 5, *A. resistens* NH1^T^.

Genome	1	2	3	4	5
Genome size (Mb)	6.0	5.0	5.9	5.0	6.1
CDS	5320	4410	5777	4263	6021
rRNA	5	4	3	5	3
tRNA	43	43	37	37	39
G+C (%)	40.1	40.9	40.4	39.3	41.9
GenBank accession	JAXRUT000000000	JAYEEY000000000	MSSW00000000	QKZU00000000	LMXN00000000

According to the Venn diagram (Fig. S4a), 2,569 core genes were shared among the novel strains and 3 related reference strains. Strains D3-2-M1T, C2-6-M1T and E1-3-M2 possessed 1,043, 360 and 516 unique genes, respectively. As strains C2-6-M1T and E1-3-M2 belonged to the same species, they shared significantly more genes (3,629) than strains C2-6-M1T and D3-2-M1T. COG and KEGG pathway analysis (levels 1 and 2, Fig. S4b, c) indicated that these newly isolated strains contained numerous genes associated with ‘Metabolism’ and ‘Environmental Information Processing’, suggesting their high metabolic activity. Within Metabolism, the most prevalent functional categories were ‘Carbohydrate Metabolism’, ‘Amino Acid Metabolism’ and ‘Metabolism of Cofactors and Vitamins’ (Fig. S4d). These results were consistent with those obtained from eggNOG and RAST analyses. There were 17% of the chromosomal genes of strain D3-2-R+10^T^ annotated by RAST and classified into 277 subsystems belonging to 27 categories. The top three subsystems were ‘Amino Acids and Derivatives’, ‘Carbohydrates’ and ‘Protein Metabolism’. Eighteen per cent of the chromosomal genes of strain C2-6-M1^T^ were annotated by RAST and classified into 270 subsystems across 27 categories. The top three subsystems were ‘Amino Acids and Derivatives’, ‘Cofactors, Vitamins, Prosthetic Groups, Pigments’ and ‘Protein Metabolism’ (Fig. S5). These results serve to evaluate the metabolic diversity and ecological potential of strains D3-2-R+10^T^ and C2-6-M1^T^, and these distributions indicate functional specialization and adaptation to different ecological niches.

### Biosynthetic gene cluster analysis

According to the antiSMASH analysis, both strains possessed a terpene gene cluster for biosynthesis of carotenoid [34], which serves as bacterial pigment protecting them from reactive oxygen species. The gene clusters of synthetizing arylpolyene in both strains showed 100% similarity to Biosynthetic Gene Cluster 0000650, annotated as the carotenoid biosynthetic gene cluster from *Algoriphagus* sp. KK10202C (Fig. S6). In the genome of strain D3-2-R+10^T^, a 7,807 bp terpene gene cluster, spanning genes 111 to 119, was located on scaffold 14. The homologous cluster in strain C2-6-M1^T^, spanning genes 40 to 46 and measuring 7,590 bp, resided on scaffold 3. This cluster contained annotated genes encoding CrtI, CrtB, CrtY, CrtD, IspH and CrtW, which were involved in carotenoid biosynthesis. Their ability for carotenoid biosynthesis is also confirmed by the special absorption peak of cell extraction at OD 450–480 nm.

### Taxonomic conclusions

To sum up, phylogenetic analysis based on 16S rRNA gene and whole-genome sequences placed strains D3-2-R+10ᵀ and C2-6-M1ᵀ within the genus *Algoriphagus*, but distinct from all validly described species. This delineation was supported by ANI, AAI and dDDH values below established species thresholds (95–96% for ANI/AAI and 70% for dDDH) compared with their closest relatives. Furthermore, physiological and biochemical characteristics differentiate the strains. Notably, they exhibit significantly higher iso-C_15 : 0_ fatty acid content than related reference strains. Therefore, strains D3-2-R+10ᵀ and C2-6-M1ᵀ represent two novel species of *Algoriphagus*. Additionally, genome analysis revealed biosynthetic gene clusters for carotenoid production in both novel strains.

## Description of *Algoriphagus aurantiacus* sp. nov.

*Algoriphagus aurantiacus* (au.ran.ti’a.cus. N.L. masc. adj. *aurantiacus*, orange coloured, referring to the colour of colonies).

Cells are Gram-stain-negative, aerobic, non-motile and rod-shaped, with 0.6–0.8 µm width and 1.2–2.4 µm in length. Colonies on MA are convex, circular, smooth, opaque and orange-pigmented after incubation at 25 °C for 3 days. Growth occurred at 4–30 °C and pH 5.0–8.5, in the presence of 0.5–13.0% (w/v) NaCl. Optimal growth was observed at 25 °C, with a pH of 7.0 and 5.0–7.0% (w/v) NaCl. No flagella and motility. Oxidase- and catalase-positive. Indole and urease were not produced. The Voges–Proskauer test was positive. Nitrate was reduced. Hydrolysed Tween 20, Tween 40 and Tween 80, but not casein, starch, sodium carboxymethylcellulose, urea and gelatin. According to the results of API 50CH strip tests, acid was produced from d-arabinose, l-arabinose, d-xylose, l-xylose, methyl-*β*-d-xylopyranoside, d-galactose, d-glucose, d-fructose, d-mannose, l-rhamnose, methyl-*α*-d-mannopyranoside, methyl-*α*-d-glucopyranoside, *N*-acetyl-glucosamine, glucosamine, amygdalin, arbutin, aesculin, salicin, d-cellobiose, maltose, d-lactose, melibiose, sucrose, trehalose, synanthrin, d-melezitose, d-raffinose, xylitol, d-gentiobiose, d-turanose, d-lyxose, d-tagatose, d-fucose and l-fucose, but not from the others. In the API ZYM test, it was positive for alkaline phosphatase, esterase (C4), esterase lipase (C8), leucine arylamidase, valine arylamidase, cystine arylamidase, trypsin, *α*-chymotrypsin, acid phosphatase, naphthol-AS-BI-phosphohydrolase, *α-*galactosidase, *β*-galactosidase, *β*-glucuronidase, *α*-glucosidase, *β*-glucosidase, *N*-acetyl-*β*-glucosaminidase and *α*-mannosidase, but negative for lipase (C14) and *β*-fucosidase. In the Biolog GEN III MicroPlate system, dextrin, d-maltose, d-trehalose, d-cellobiose, gentiobiose, sucrose, turanose, stachyose, d-raffinose, *α*-d-lactose, d-melibiose, *β*-methyl-d-glucoside, d-salicin, *N*-acetyl-d-glucosamine, *N*-acetyl-*β*-d-mannosamine, *N*-acetyl-d-galactosamine, *α*-d-glucose, d-mannose, d-fructose, d-galactose, l-fucose, d-sorbitol, d-mannitol, d-arabitol, d-glucose-6-PO_4_, glycyl-l-proline, l-arginine, l-aspartic acid, l-glutamic acid, l-serine, pectin, d-gluconic acid, glucuronamide, l-lactic acid, l-malic acid, d-galacturonic acid and *γ*-amino-butyric acid were utilized. In the API 20NE and 20E tests, positive for nitrate reduction, glucose fermentation and hydrolysis of *α*-glucosidase and *β*-galactosidase. In the Voges–Proskauer test, negative for arginine dihydrolase, lysine decarboxylase, ornithine decarboxylase, urease, tryptophane deaminase and hydrolysis of gelatin. The major quinone was MK-7. The predominant polar lipids included PE, one PL and five unidentified lipids (L1–L5). The primary cellular fatty acids of strains were iso-C_15 : 0_, summed feature 3 (C_16 : 1_* ω7*c*/*C1_6 : 1_* ω6*c) and summed feature 9 (iso-C_17 : 1_* ω9*c*/*C1_6 : 0_ 10-methyl). The strain resistant to penicillin (10 IU), tobramycin (10 µg), netilmicin (30 µg), ceftriaxone (30 µg), cefotaxime (30 µg), cefuroxime sodium (30 µg), minocycline (30 µg), sulfamethoxazole/trimethoprim (1.25 µg), amikacin (30 µg), ceftazidime (30 µg), cephalotin (30 µg) and oxacillin (1 µg).

The type strain D3-2-R+10^T^ (CGMCC 1.62672^T^=KCTC 102180^T^) was isolated from the shore soil of LungmuCo Lake in Xizang Autonomous Region, China. The genomic DNA G+C content of the type strain was 40.1%. The GenBank/EMBL/DDBJ accession numbers for the 16S rRNA gene sequence and genome were OR880264 and JAXRUT000000000, respectively.

## Description of *Algoriphagus persicinus* sp. nov.

*Algoriphagus persicinus* (per.si.ci’nus. N.L. masc. adj. *persicinus*, peach coloured, referring to the colour of colonies).

Cells are Gram-stain-negative, aerobic, non-motile and rod-shaped, 0.6–0.8 µm in width and 1.2–2.2 µm in length. Colonies on MA are convex, circular, smooth, opaque and pink-pigmented after incubation at 25 °C for 3 days. Growth occurred at 4–30 °C and pH 5.0–8.0, in the presence of 0.5–15.0% (w/v) NaCl. Optimal growth was observed at 20 °C, with a pH of 7.0 and 5.0–7.0% (w/v) NaCl. No flagella and motility. Oxidase- and catalase-positive. Indole and H_2_S were not produced. The Voges–Proskauer test was negative. Nitrate was reduced to nitrite. Hydrolyses Tween 20 and Tween 80, but not casein, starch, sodium carboxymethyl cellulose, urea and gelatin. According to the results of API 50CH strip tests, acid was produced from d-arabinose, l-arabinose, d-xylose, l-xylose, methyl-*β*-d-xylopyranoside, d-galactose, d-glucose, d-fructose, d-mannose, l-rhamnose, methyl-*α*-d-mannopyranoside, methyl-*α*-d-glucopyranoside, *N*-acetyl-glucosamine, amygdalin, arbutin, aesculin, salicin, d-cellobiose, maltose, d-lactose, melibiose, sucrose, trehalose, synanthrin, d-melezitose, d-raffinose, xylitol, d-gentiobiose, d-turanose, d-lyxose, d-tagatose, d-fucose and l-fucose, but not from others. In the API ZYM test, it was positive for alkaline phosphatase, esterase (C4), esterase lipase (C8), leucine arylamidase, valine arylamidase, cystine arylamidase, trypsin, *α*-chymotrypsin, acid phosphatase, naphthol-AS-BI-phosphohydrolase, *α-*galactosidase, *β*-galactosidase, *β*-glucuronidase, *α*-glucosidase, *β*-glucosidase, *N*-acetyl-*β*-glucosaminidase and *α*-mannosidase, but negative for lipase (C14) and *β*-fucosidase. In the Biolog GEN III MicroPlate system, dextrin, d-maltose, d-trehalose, d-cellobiose, gentiobiose, sucrose, turanose, stachyose, d-raffinose, *α*-d-lactose, d-melibiose, *β*-methyl-d-glucoside, d-salicin, *N*-acetyl-d-glucosamine, *N*-acetyl-*β*-d-mannosamine, *N*-acetyl-d-galactosamine, *α*-d-glucose, d-mannose, d-fructose, d-galactose, d-fucose, l-fucose, l-rhamnose, d-sorbitol, d-mannitol, d-arabitol, d-glucose-6-PO_4_, d-serine, glycyl-l-proline, l-arginine, l-aspartic acid, l-glutamic acid, l-serine, pectin, d-gluconic acid, glucuronamide, l-lactic acid, l-malic acid, l-alanine, d-glucuronic acid, acetoacetic acid, propionic acid, glycerol, formic acid and acetic acid were utilized. In the API 20NE and 20E tests, positive for glucose fermentation and hydrolysis of *α*-glucosidase and *β*-galactosidase and negative for arginine dihydrolase, lysine decarboxylase, ornithine decarboxylase, urease, tryptophane deaminase and hydrolysis of gelatin. The predominant quinone was MK-7. The major polar lipids included PE, two PLs (PL1–PL2) and seven unidentified lipids (L1–L7). The main cellular fatty acids were iso-C_15 : 0_, summed feature 3 (C_16 : 1_* ω7*c*/*C1_6 : 1_* ω6*c) and summed feature 9 (iso-C_17 : 1_* ω9*c*/*C1_6 : 0_ 10-methyl). Resistant to tobramycin (10 µg), netilmicin (30 µg), ceftriaxone (30 µg), cefazolin (30 µg), cefotaxime (30 µg), cefuroxime sodium (30 µg), sulfamethoxazole/trimethoprim (1.25 µg), amikacin (30 µg), ceftazidime (30 µg), cephalotin (30 µg) and oxacillin (1 µg).

The type strain C2-6-M1^T^ (CGMCC 1.62677^T^=KCTC 102179^T^) was isolated from the shore soil of LungmuCo Lake in Xizang Autonomous Region, China. The genomic DNA G+C content of the type strain was 40.9%. The GenBank/EMBL/DDBJ accession numbers for the 16S rRNA gene sequence and the genome of strain C2-6-M1^T^ were PP150897 and JAYEEY000000000, respectively.

## Supplementary material

10.1099/ijsem.0.006826 Uncited Supplementary Material 1.

## Abbreviations

AAI: average aa identity
ANI: average nt identity
CDS: coding sequence
CIS: contractile injection system
COG: clusters of orthologous group
dDDH: digital DNA–DNA hybridization
L: unidentified polar lipid
MA: marine 2216 agar
MB: marine 2216 broth
PE: phosphatidylethanolamine
PL: unidentified phospholipid
RAST: Rapid Annotation using Subsystem Technology
