# Supplementary material for: Algoriphagus aurantiacus sp. nov. and Algoriphagus persicinus sp. nov., two novel species isolated from the shore soil of salt lake
Source: Int J Syst Evol Microbiol. 2025 Jun 27;75(6):006826. doi: 10.1099/ijsem.0.006826 (PMC12205151; doi:10.1099/ijsem.0.006826)
Supplement: Uncited Supplementary Material 1. [file ijsem-75-06826-s001.pdf]

Algoriphagus aurantiacus sp. nov. and *Algoriphagus persicinus* sp. nov., two novel species isolated from shore soil of salt lake

Yan-Yan Zheng[1], Xuan Zhang[2,4], Zi-Xuan Liu[2], Rui Wang[3], Dorji Phurbu[1], Ai-Hua Li[2*]

1. Xizang Key Laboratory of Plateau Fungi, Institute of Plateau Biology of Xizang Autonomous Region, Lhasa, Xizang Autonomous Region 850001, PR China;
2. China General Microbiological Culture Collection Center, Institute of Microbiology, Chinese Academy of Sciences, Beijing 100101, PR China;
3. Tianjin Institute of Industrial Biotechnology, Chinese Academy of Sciences, Tianjin 300308, PR China;
4. School of Biotechnology and Food Science, Tianjin University of Commerce, Tianjin, PR China.

Correspondence: Ai-Hua Li, liah@im.ac.cn; Tel: 86-01-64806073

Keywords: *Algoriphagus*; polyphasic taxonomy; genome; salt lake; Xizang

Author Notes: The GenBank/EMBL/DDBJ accession numbers for the 16S rRNA gene of strains D3-2-R+10^T, C2-6-M1^T and E1-3-M2 were OR880264, PP150897 and PP150898, respectively. The Whole Genome Shotgun projects of strains D3-2-R+10^T, C2-6-M1^T and E1-3-M2 have been deposited at DDBJ/ENA/GenBank under the assembly accession numbers JAXRUT000000000, JAYEEY000000000 and JAXUHY000000000, respectively.

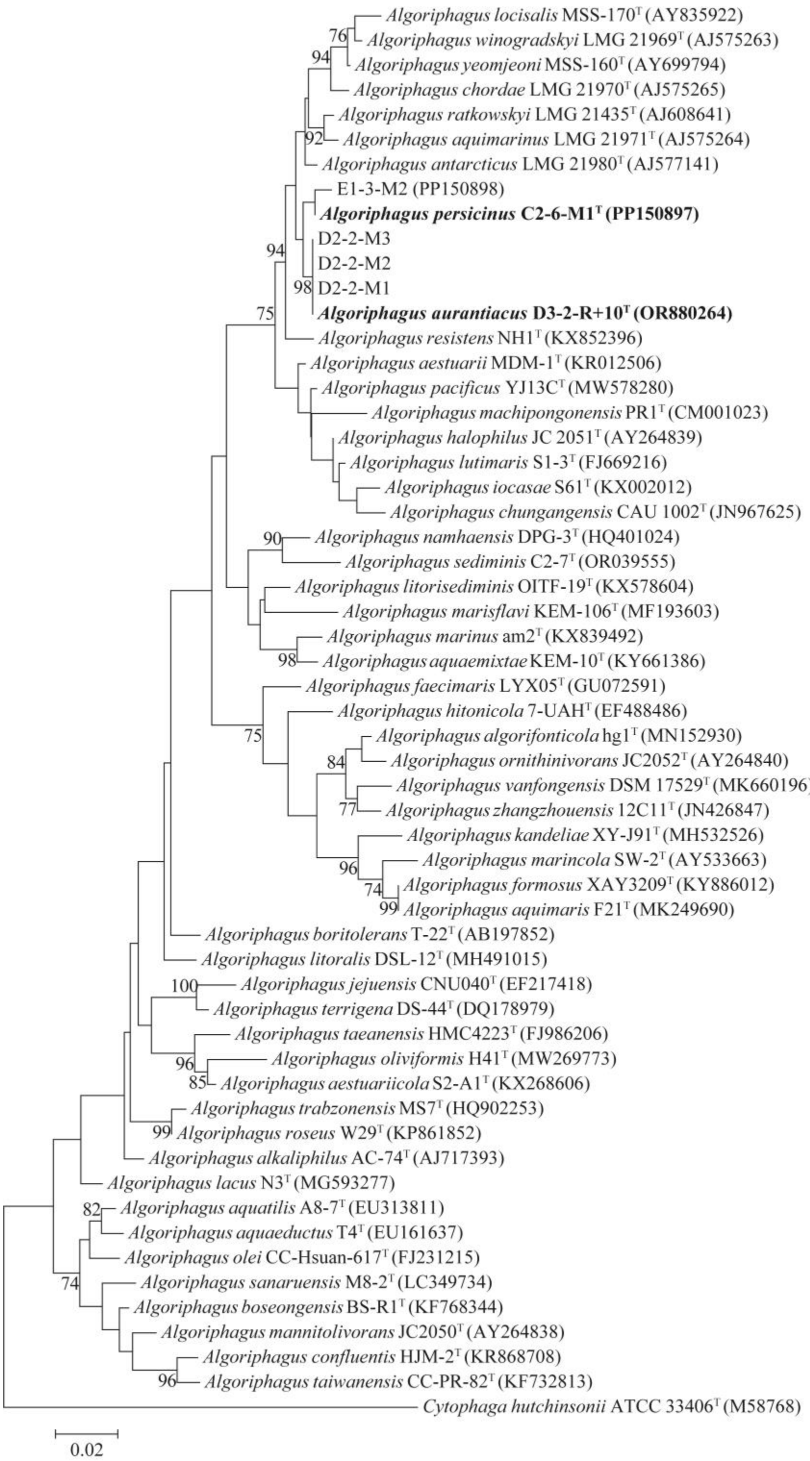

Fig. S1. Evolutionary relationships between strains D3-2-R+10^T, C2-6-M1^T, E1-3-M2, D2-2-M3, D2-2-M2, D2-2-M1 and species of the genus *Algoriphagus* based on 16S rRNA gene sequences, inferred using the maximum-likelihood algorithm. Bootstrap values higher than 70% are shown (percentages of 1,000 replications) on the branches. Cytophaga hutchinsonii LMG 10844^T was used as an outgroup. Bar, 0.02 changes per nucleotide position.

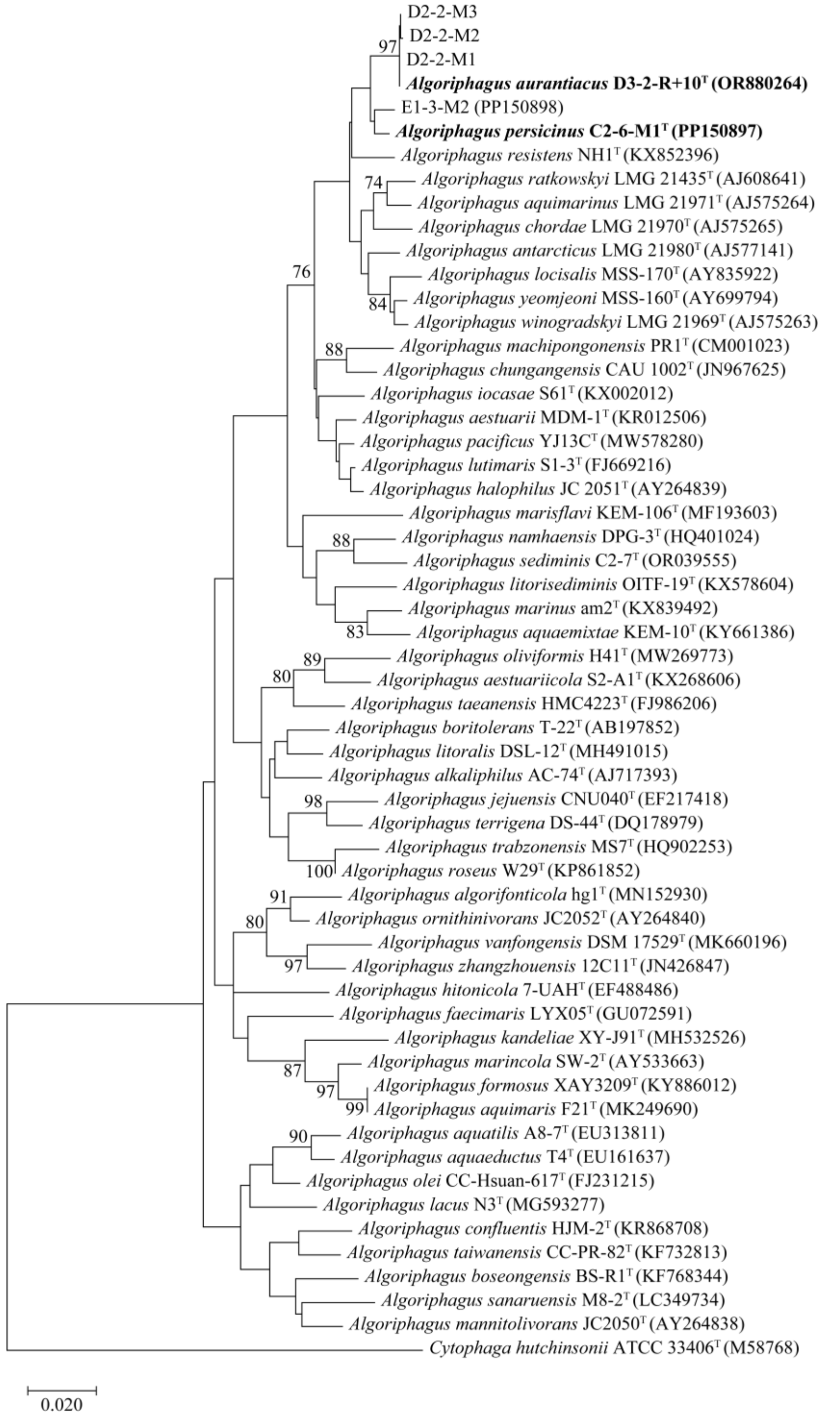

Fig. S2. Evolutionary relationships between strains D3-2-R+10^{T}, C2-6-M1^{T}, E1-3-M2, D2-2-M3, D2-2-M2, D2-2-M1 and species of genus *Algoriphagus* based on 16S rRNA gene sequences, inferred using the minimum-evolution algorithm. Bootstrap values higher than 70% are shown (percentages of 1,000 replications) on the branches. Cytophaga hutchinsonii LMG 10844T was used as an outgroup. Bar, 0.02 changes per nucleotide position.

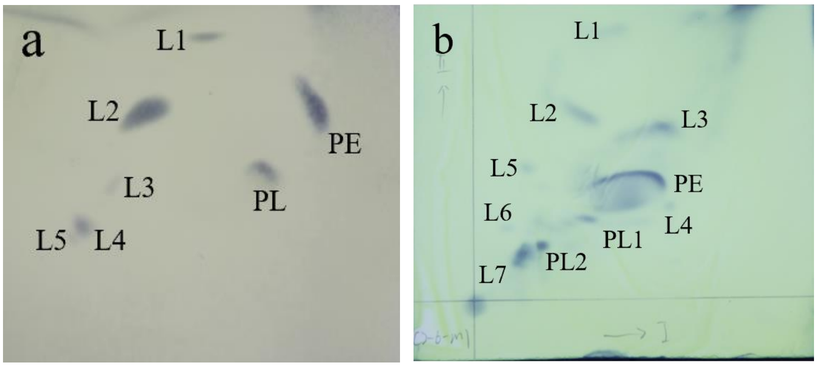

Fig. S3. Polar lipids profiles of strains D3-2-R+10^{T} (a) and C2-6-M1^{T} (b) separated by two-dimensional TLC, which were detected by spraying with molybdatophosphoric acid reagent. PE, phosphatidylethanolamine; PL, unidentified phospholipid; L, unidentified lipid.

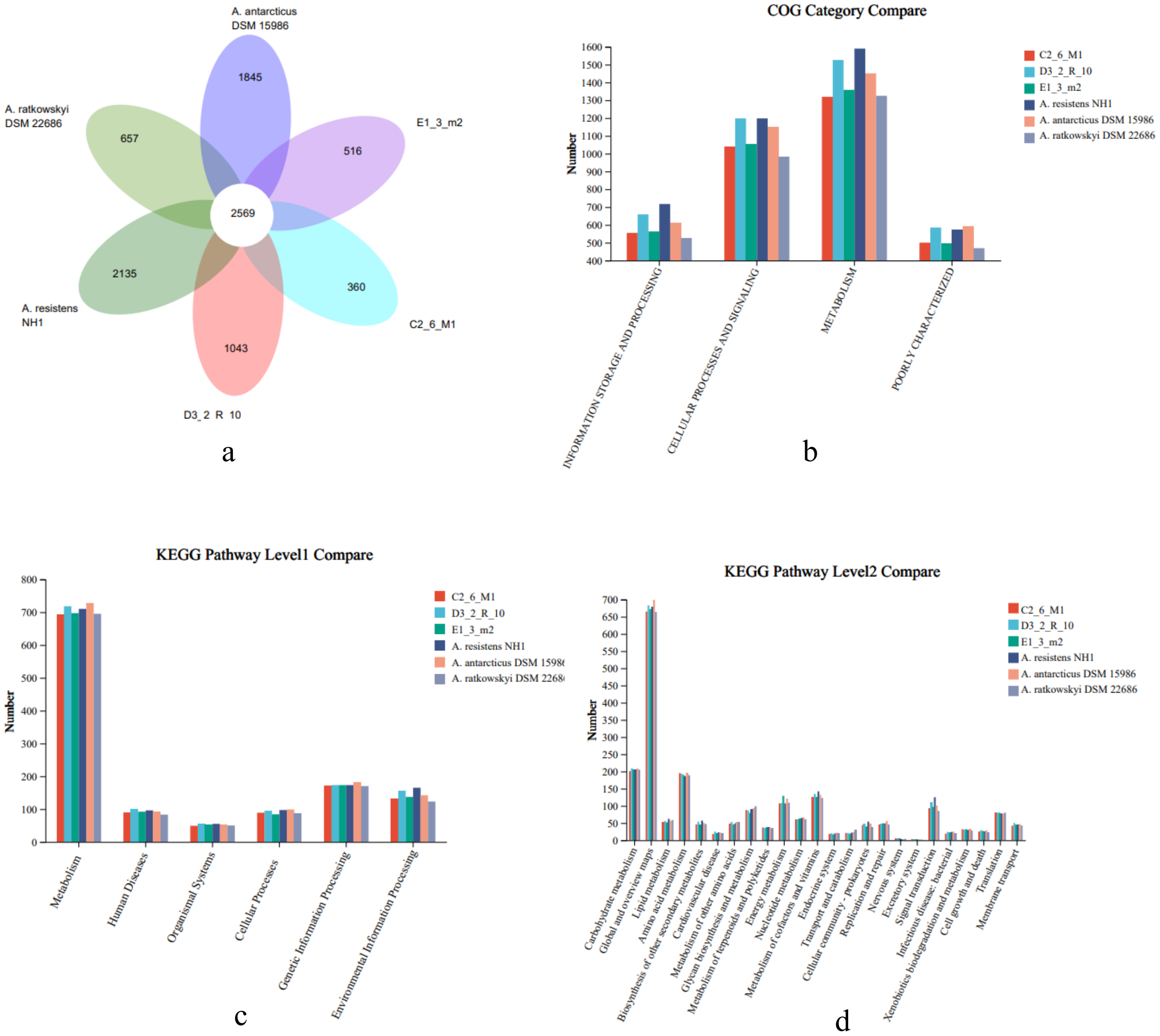

Figure S4. Venn diagrams (a) and Genome annotation by COG (b) and KEGG (c, d).

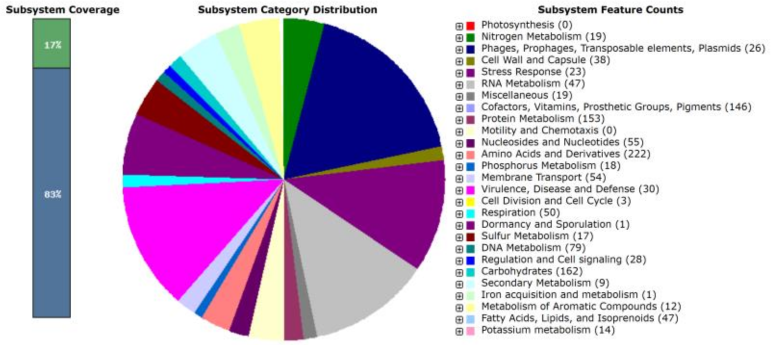

a

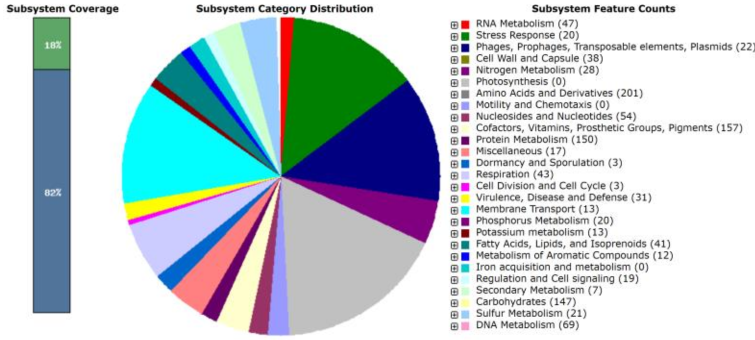

b

Fig. S5. Genome annotation of strains D3-2-R+10^T (a) and C2-6-M1^T (b) using the RAST server. Green part in bar chart corresponds to percentage of proteins included. Pie chart and count of subsystem features in right panel show percentage distribution and category of subsystems in strains D3-2-R+10^T and C2-6-M1^T.

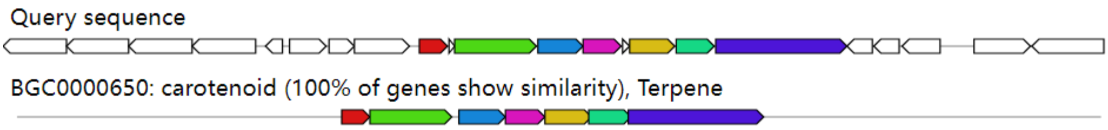

a

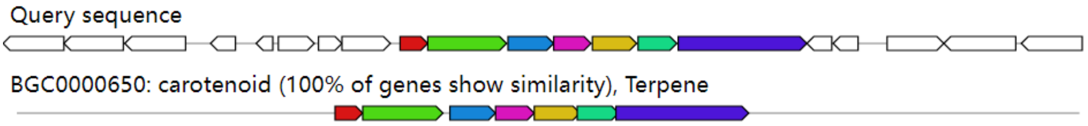

b

Fig. S6. Gene clusters for biosynthetic of carotenoid appeared in strains D3-2-R+10^T (a) and C2-6-M1^T (b).

Table S1. Antibiotic sensitivity test for stains D3-2-R+10^T and C2-6-M1^T. Strains: 1, D3-2-R+10^T; 2, C2-6-M1^T. S, sensitive; M, moderate resistance; R, resistant.

Antibiotics	1	2
fleroxacin (5 μg)	S	S
lomefloxacin (10 μg)	S	S
cipronfloaxacin (5 μg)	S	S
penicillin (10 IU)	R	M
erythromycin (15 μg)	S	S
chloramphenicol (30 μg)	S	S
azithromycin (15 μg)	S	S
clindamycin (2 μg)	S	S
doxycycline (30 μg)	M	M
clarithromycin (15 μg)	S	S
tobramycin (10 μg)	R	R
vancomycin (30 μg)	S	S
netilmicin (30 μg)	R	R
ceftriazone (30 μg)	R	R
cefaclor (30 μg)	M	S
cefazolin (30 μg)	M	R
cefotaxine (30 μg)	R	R
ampicillin (10 μg)	M	M
cefurosimc sodium (30 μg)	R	R
minocycline (30 μg)	R	S
rifampin (5 μg)	S	S
tetracycline (30 μg)	M	S
sulfamethoxazole/trimethoprim (1.25 μg)	R	R
amikacin (30μg)	R	R
ceftazidime (30 μg)	R	R
cephalotin (30 μg)	R	R
cefoperazone (75 μg)	M	S
piperacillin (100 μg)	S	S
oxacillin (1 μg)	R	R
nitrofurantoin (300 μg)	M	S
